# Predicting the Effect of Localized ACL Damage on Neighbor Ligament Mechanics via Finite Element Modeling

**DOI:** 10.3390/bioengineering9020054

**Published:** 2022-01-28

**Authors:** Alexander Knapp, Lakiesha N. Williams

**Affiliations:** Department of Biomedical Engineering, University of Florida, Gainesville, FL 32608, USA; a.knapp1993@ufl.edu

**Keywords:** ACL, tissue mechanics, continuum damage mechanics

## Abstract

The anterior cruciate ligament (ACL) plays a pivotal role in support of the knee under loading. When damaged, it is known that substantial changes in the mechanics of the neighboring ligaments can be observed. However, a localized damage approach to investigating how ACL deficiency influences the neighboring ligaments has not been carried out. To do this, a finite element model, incorporating a continuum damage material model of the ACL, was implemented. Localized ACL damage was induced using high quadriceps force loading. Once damaged, anterior shear forces or tibial torque loadings were applied to the knee joint. The relative changes in stress contour and average mid-substance stress were examined for each of the neighboring ligaments following localized ACL damage. It was observed that localized ACL damage could produce notable changes in the mechanics of the neighboring knee ligaments, with non-homogenous stress contour shape changes and average stress magnitude being observed to increase in most cases, with a notable exception occurring in the MCL for both loading modes. In addition, the ligament bearing the most loading also changed with ACL deficiency. These changes carry implications as to morphological effects that may be induced following localized ACL damage, indicating that early diagnosis of ACL injury may be helpful in mitigating other complications post injury.

## 1. Introduction

The anterior cruciate ligament (ACL) is a major structural component of the knee, being primarily responsible for inhibiting anterior tibial translation but also playing a role in supporting the knee during valgus, varus, and torque moments [1,2,3,4]. Once torn, patients report high pain values and noticeable increases in joint instability, with potentially high medical costs for treatments such as surgical intervention [5,6]. Once damaged, the rehabilitation of the ACL is difficult due to poor vascularization within the knee joint. Consequentially, long-term lifestyle changes can occur, which can be particularly detrimental to athletic careers, leading to long-term or even lifelong removal from play [7,8,9]. Due to the prevalence and potential severity of complications following injury, it is paramount to increase our understanding of the mechanisms of ACL injury and resulting joint kinematic changes. With increased knowledge of the mechanics of ACL injury, clinicians and athletic trainers can devise new methods to prevent ACL injury.

The ACL performs this function in concert with its neighboring ligaments, the posterior cruciate, lateral collateral, and medial collateral ligaments (PCL, LCL, and MCL, respectively). Kwon et al. and DeMorat et al. experimentally showed that once the ACL is torn, the kinematics of the knee change significantly, with increases in tibial translation and rotation of up to 100% [10,11]. Li et al. modeled the effects of complete ACL mechanical property degradation, observing even greater increases in motion than Kwon et al. and DeMorat et al. [12]. Rahemi et al. saw similar increases as well, noting that the degree of change was dependent on which bundles(s) of the ACL were torn [13]. Using microstructural analysis and computational simulations, the work of Ochi et al. and Moglo et al. indicated that a decrease in PCL properties leads to microstructural changes in the ACL, as well as significant increases in ACL strain with deficient PCL ligaments [14,15] Similarly, Lujan et al. found that strains in the MCL can increase up to 4% as ACL deficiency occurs [16]. Consistently, in all the aforementioned works the change in mechanics vary based on the loading condition of the knee. By altering the loading carried by the neighboring ligaments after ACL damage, stress shielding effects may occur, ultimately altering the morphology of the tissue. Consequentially, due to shifts in the mechanics of the other ligaments post injury, there is an increased likelihood of additional complications, such as MCL and PCL tearing. This carries implications as to the need to prevent ACL injury, such that these additional injuries do not occur.

These findings obtained via numerical simulation were all based on models that assumed the ACL had homogenous damage. This methodology neglects the possibility of non-unform and partial tears within the tissue. It has been established via Noyes and Torvik’s work. that the ACL often tears in a smooth continuous process [17]. Similar behavior has been observed by Panjabi et al. and Provenzano et al., for both the ACL and MCL [18,19] Altered fibers, post damage, can play a major role in the progression of localized damage [20]. That is, it is possible for localized damage to happen within the tissue without rapid failure throughout the ligament. By modeling mechanical degradation in the ACL as homogenous, historically modeling studies have overestimated the joint kinematic changes that may occur due to damage. The goal of the present work is to investigate how localized damage induced via different loading types changes the mechanics of the neighboring ligaments. To show this, injury will be induced to the ACL in a finite element model of the knee under anterior shear loading. The changes in the mechanics in subsequent reloading in anterior shear and internal torsion will be examined.

## 2. Materials and Methods

### 2.1. Damage Modeling Procedure

A modified Helmholtz free energy approach was used to model damage within the ACL. First, for a known fiber orientation *a*_0_, an anisotropic strain energy density form was assumed for the non-damage behavior using the incompressible Holzapfel model, as in Equation (1), where the strain energy density is a combination of isotropic, anisotropic, and volumetric components, each a function of the invariants of the right Cauchy–Green deformation tensor *C* ($I_{1}=trace\left(C\right),\;I_{4}=\sqrt{a_{0}C\cdot a_{0}},\;J=\det\left(C\right)$) [21].

(1) $$W=C_{1}\left(I_{1}-1\right)+\frac{k_{1}}{2k_{2}}\left(e^{k_{2}{\left(k\left(I_{1}-3\right)+\left(1-3k\right)\left(I_{4}-1\right)\right)}^{2}}-1\right)+\frac{1}{D}\left(\frac{J^{2}-1}{2}-\ln\left(J\right)\right)$$

where *C*_1_, *k*_1_, *k*_2_ and *D* are material constants, and *k* is the fiber dispersion parameter assumed to have a value of zero in this work, meaning that the fibers are fully aligned. This is reasonable based on the microscopy results from Mclean et al., fiber alignment being distributed normally around a singular value [22]. Assuming near incompressibility, the volumetric term becomes negligible. To model damage, the strain energy density formulation was modified using a similar approach as Natali et al. [23,24], such that

(2) $$W_{modified}=G\left(\lambda_{max}\right)*\left(W\right)$$

where

(3) $$G(\lambda_{max})=\frac{1-e^{\beta^{2}\left(\lambda_{max}^{4}-\lambda_{c}^{4}\right)}}{1-e^{\beta^{2}(\lambda_{0}^{4}-\lambda_{c}^{4})}}$$

In this approach, *λ*_0_ is a fiber stretch where damage initiates, and *λ_c_* is the fiber stretch wherein the tissue is completely torn. *β* is a parameter that indicates the rate of damage. Assuming the fibers were uniformly distributed in the loading direction, the material parameters were developed using the least squared error best fit of the average stress strain data of all the knee ligaments from Ristaniemi et al. to the chosen form, allowing for an approach capable of capturing the mechanical degradation of the ACL tissue as the fiber stretch increases [25]. For practical modeling purposes, a degradation limit of 25% of the undamaged parameters was implemented in the model. The material constants are summarized in Table 1.

This material model was then incorporated into the finite element modeling software, ABAQUS (Dassault, Vélizy-Villacoublay, France), using the user-defined field subroutine [26]. The remaining connective ligaments were also modeled using this average stress strain data with the same mechanical property parameters, sans damage. To model the bones, a Young’s modulus of E = 20 GPa with an assumed nearly incompressible Poisson’s ratio of *v* = 0.42 from Rupin et al., was used [27].

### 2.2. Model Setup

The model geometry (tibia, femur, fibula, ACL, MCL, PCL, LCL) was developed using images courtesy of the National Institutes of Health’s Visible Human Project for creating the Finite Element Model for the study [28]. Using these images, each tissue was edited using thresholding techniques and stacked using the visualization software ImageJ [29]. The ACL was modeled with damage capabilities using the user-defined field subroutine in ABAQUS, while the neighboring ligaments were given the same material properties sans damage modifications. Fiber orientations were assigned as in the work of Wan et al., by assigning one fiber direction by connecting one end of the ligament to the other [30,31]. In addition, the meniscus was not modeled in this analysis, as preliminary tests indicated it had minimal effect on the loading cases examined in this study. All geometry was meshed using modified tetrahedral elements, the modified elements being chosen to eliminate the possibility of volumetric locking, with a total mesh size of approximately 1.5 million nodes.

**Figure 1:**
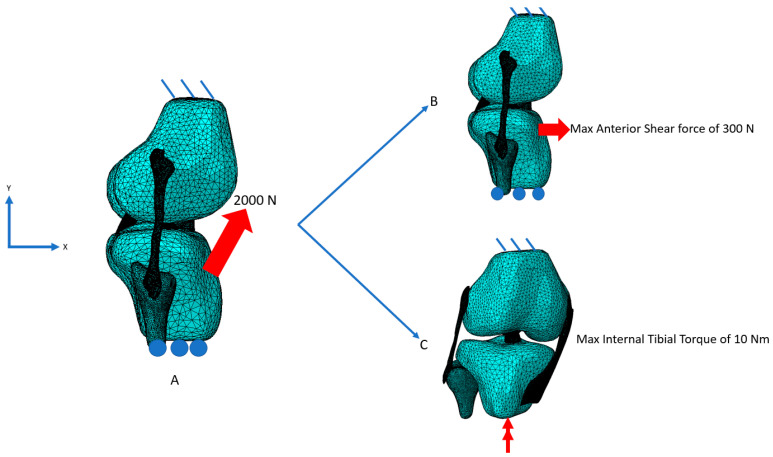
Illustration of loading and boundary conditions for damage initiation and subsequent examination. (**A**) To initiate damage, a quadriceps force loading of 2000 N was applied and removed. (**B**) Following damage initiation, an anterior shear force of up to 300 N was applied, or (**C**) a tibial torque up to 10 Nm was applied.

The following procedure was performed to examine the change in loading under anterior shear and tibial torque within the MCL, PCL, and LCL, following localized ACL damage. First, to establish a baseline for comparison, the average mid-substance von Mises stress was recorded in each tissue for non-damaged ACL tissue. This was performed for anterior shear forces of up to 300 N and tibial torques of up to 10 Nm, while keeping the tibia fixed in the y axis and the femur and top of each ligament fixed in all directions (Figure 1A–C) in a similar manner to Wan et al. and Kwon et al. by applying the anterior shear force through the middle of the horn region, while the tibial torque as applied as a force couple at the end of the tibia [10,30]. Each ligament was attached to the tibia via tie constraints.

**Figure 2:**
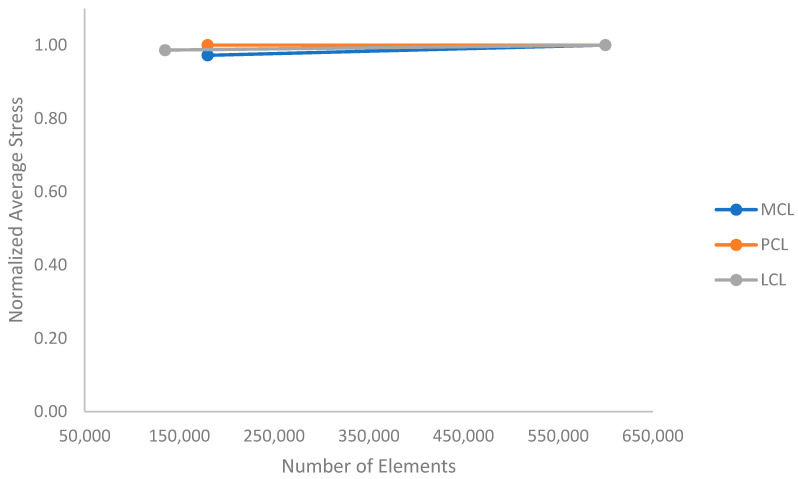
Convergence tests for MCL, PCL, and LCL meshes. The maximum percent difference between data points is less than 3% with an element increase of 500,000. Therefore, the meshes are converged.

Secondly, to induce a state of moderate localized damage, a high quadriceps tendon loading force of 2000 N was applied and then removed, as shown in Figure 1A. Following this, an anterior shear force of up to 300 N (Figure 1B) or an internal tibial torque of up to Nm was applied (Figure 1C). These loadings were chosen as they correspond to common injury mechanisms of the ACL. Third and finally, the loading procedures of Figure 1B and Figure 2C were performed with the ACL removed to examine the effects of complete ACL destruction. The average results and contour plots of the mid-substance von Mises stress were recorded in the MCL, PCL, and LCL for each step of this procedure. Here the von Mises stress was chosen so as to give results in an average sense that also includes potential changes caused by changing shear stresses perpendicular to the fiber direction, which, in conjunction with the stress in the fiber direction, can lead to morphological changes in the tissue. To compare with literature, the contour plots for the anterior shear loadings were taken at 100 N, while the torque loadings were examined at 10 Nm.

Convergence studies were performed on the PCL, MCL, and LCL geometry using average stresses in the mid-substance regions, the results of which can be seen in Figure 2. As can be seen, each tissue mesh has converged, with the maximum percent difference between data points of less than 3%.

## 3. Results

**Figure 3:**
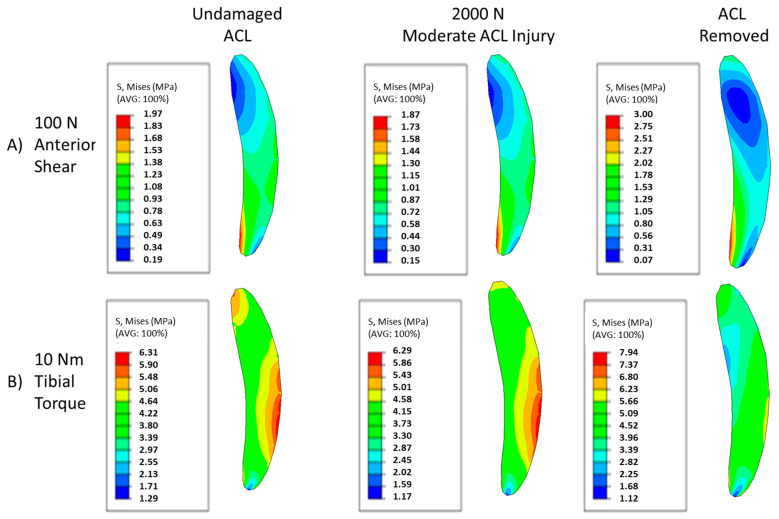
Contour plots of MCL mid-substance von Mises stress (Pa). (**A**) Contour results for at 100 N anterior shear force on the tibia for undamaged, moderate injury, and complete ACL destruction. (**B**) Contour results for at 10 Nm tibial torque on the tibia for undamaged, moderate injury, and complete ACL destruction.

The changing von Mises stress contour patterns in the mid substance of the MCL for both anterior shear and tibial torque loadings can be seen in Figure 3. Moderate injuries caused by the 2000 N quadriceps force can induce subtle shifts in the contour, and complete destruction of the ACL yields drastically different stress contours. As damage increases, the minima in the posterior region of the MCL shifts anteriorly in the loading case of anterior shear (Figure 3A). Examining Figure 3B, moderate levels of injury yield subtle shifts in the von Mises contour plot for torque loadings, with the contour remaining largely the same pattern, with minor shifts occurring in the posterior region of the tissue. However, once the ACL is fully torn, the distribution becomes more homogenous and primarily composed of lower stresses than the lower damage cases. For both loading cases, the loading on the MCL has fundamentally changed.

**Figure 4:**
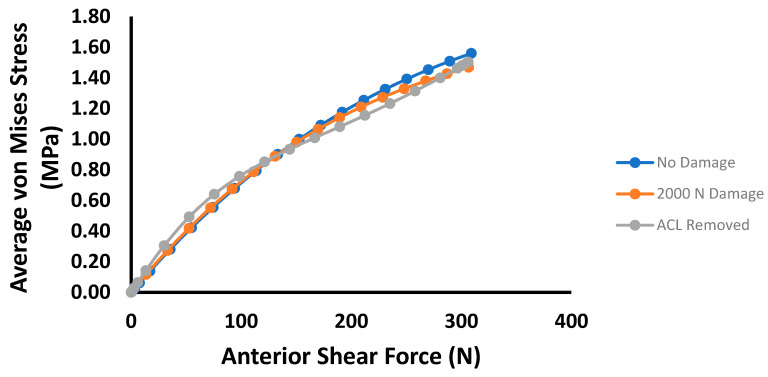
Plot of average MCL mid-substance von Mises stress (MPa) versus anterior shear force for the three prescribed damage cases.

**Figure 5:**
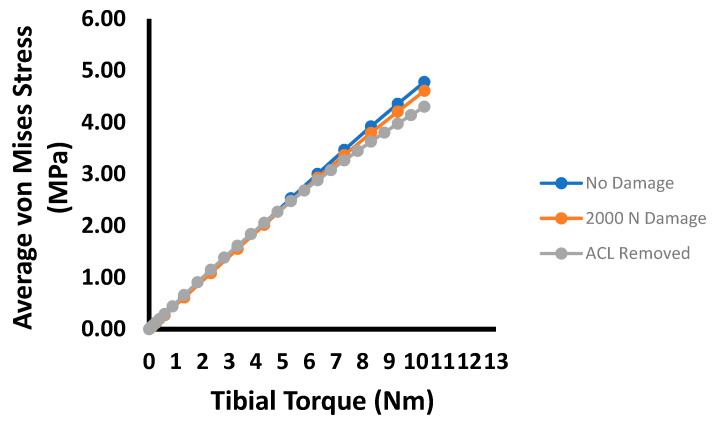
Plot of average MCL mid-substance von Mises stress (MPa) versus internal tibial torque for the three prescribed damage cases.

The average von Mises stresses for each damage case in the mid-substance are shown in Figure 4. Up to 100 N, the overall average von Mises stress increases as damage in the ACL develops. However, this trend does not correspond to the maximum stress in the cross-section. Instead, the maximum stress decreases slightly and eventually increases as damage in the ACL expands. Similarly, Figure 5 is the von Mises stress for each damage case for the tibial torque loading case, predicting lower average mid-substance stress levels as damage increases. Prior to 6 Nm, very slight increases in stress occur; however, these are likely not significant enough to be of any practical value. Beyond 6 Nm, greater pronounced changes occur in the average mid-substance stress, with the stress carried decreasing as damage develops.

**Figure 6:**
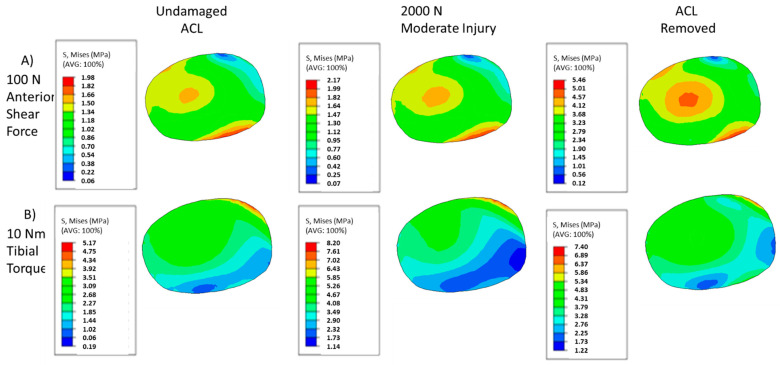
Contour plots of PCL mid-substance von Mises stress (MPa). (**A**) Contour results for at 100 N anterior shear force on the tibia for undamaged, moderate injury, and complete ACL destruction. (**B**) Contour results for at 10 NM tibial torque on the tibia for undamaged, moderate injury, and complete ACL destruction.

The von Mises stresses of the PCL mid-substance for the prescribed damage scenarios and loading cases are shown in Figure 6. As with the MCL, the amount of damage can have subtle and significant impacts on the stress contour, with both the pattern and local maxima changing with increased damage for each loading case. Figure 6A illustrates the von Mises contour plots in the PCL mid-substance for each damage case examined. Most prominently, a stress riser appears to develop as the ACL is damaged, with a significant increase in stress occurring in the posterior part of the cross-section. At the same time, the high stresses on the anterior end become more concentrated. In addition, the low stress regions reduce in size as the stress riser increases in the middle of the tissue. Unlike in the MCL contour, the maximum stress observed increases continuously as damage develops in the ACL.

Similar overall trends in the PCL, compared to the anterior shear case, can be observed in torque loading as indicated by Figure 6B. As damage increases, the general shape of the contour stays roughly the same, with regions of lower stress becoming more pronounced. In addition, as with the anterior shear case in Figure 6A, a stress riser appears to develop as damage increases under torque loading, though this riser is less pronounced than its anterior shear counterpart. It can also be observed that the maximum stresses do not consistently increase, though in average they do, implying non-homogenous increases in stress within the tissue.

**Figure 7:**
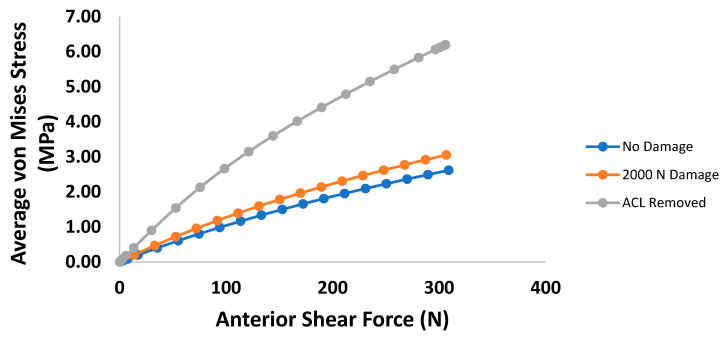
Plot of average PCL mid-substance von Mises stress (MPa) versus anterior shear force for the three prescribed damage cases.

**Figure 8:**
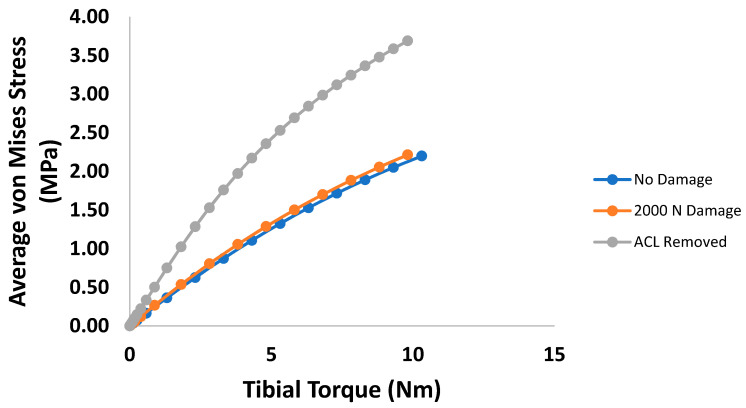
Plot of average PCL mid-substance von Mises stress (MPa) versus internal tibial torque for the three prescribed damage cases.

Examining the average mid-substance von Mises stress results of the anterior shear case within the PCL, the changes caused by induced damage always increases the stress carried, with drastic changes occurring with a fully torn ACL (Figure 7). Similarly, Figure 8 shows the average PCL mid-substance von Mises stress for varying damage levels for the torque loading case. On average, the stress in the PCL for the torque loading case increases with damage, as indicated in Figure 8. For moderate injuries, only slight increases in stress were observed. However, for a fully torn ACL, the stress in the PCL increased greatly.

**Figure 9:**
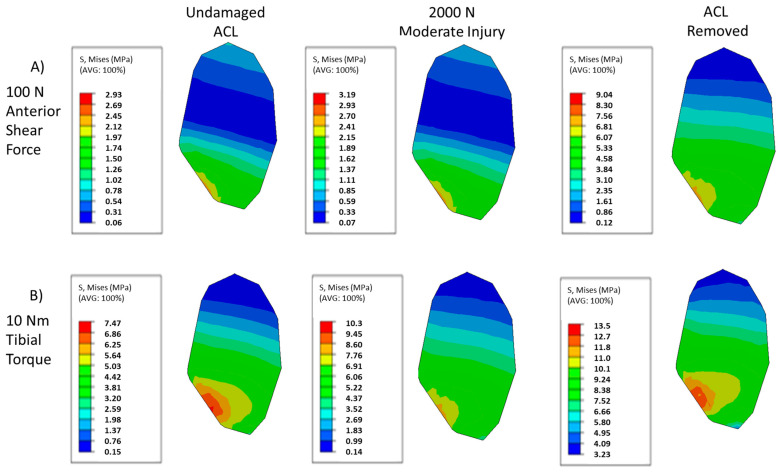
Contour plots of LCL mid-substance von Mises stress (MPa). (**A**) Contour results for at 100 N anterior shear force on the tibia for undamaged, moderate injury, and complete ACL destruction. (**B**) Contour results for at 10 Nm tibial torque on the tibia for undamaged, moderate injury, and complete ACL destruction. In each loading case, the contour changes as damage is induced.

Figure 9 collects the mid-substance von Mises stress contour plots for the LCL for the damage and loading cases prescribed for this study. Examining Figure 9A, similar trends compared to the other ligaments under anterior shear loading can be observed within the LCL in the von Mises contour plot as damage increases. As the damage in the ACL increases, the high stress regions of the LCL contour plot appear to spread out in the posterior direction, causing the low stress regions to become comparatively smaller. Though a stress concentration can be observed near the anterior edge, this stress riser does not propagate as prominently as in the PCL, staying largely confined to the edge while the surrounding stresses increase in the posterior direction. The maximum stresses can be observed to always increase. Figure 9B shows a similar behavior in torque loading within the LCL when compared to the anterior shear case. The high stress regions increase in the posterior direction as localized ACL damage increases. Interestingly, the highest stress region becomes more similarly scaled to its surroundings, before ultimately increasing further as in the fully torn ACL case. However, the maximum stress continues to increase with increased localized ACL damage, despite the shifts in the relative values of the contour plot.

**Figure 10:**
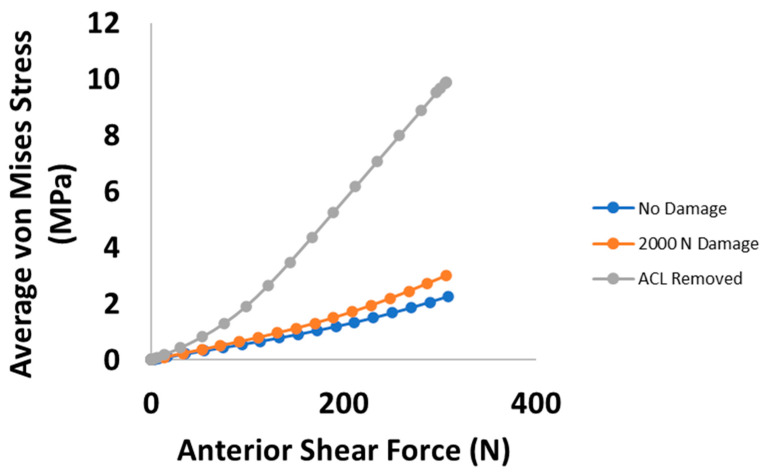
Plot of average LCL mid-substance von Mises stress (MPa) versus anterior shear force for the three prescribed damage cases.

**Figure 11:**
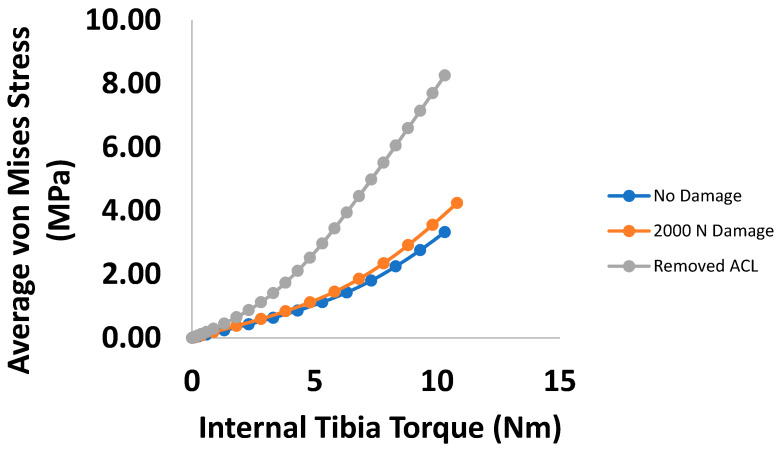
Plot of average LCL mid-substance von Mises stress (MPa) versus internal tibial torque for the three prescribed damage cases.

As with the PCL, as ACL damage increases, the differences in average von Mises stress begin subtly and become much more pronounced when the ACL is fully torn (Figure 10). Figure 11 yields similar trends in the torque case for stress within the LCL as in the anterior shear case. It can be observed that for moderate damage levels, relatively small increases in stress within the LCL occur, with large changes occurring in the fully damaged case.

**Figure 12:**
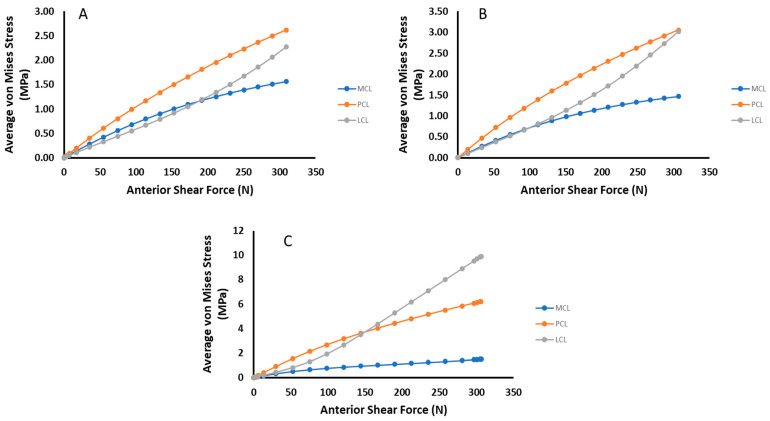
Comparative plots of the relative stress amounts for each ligament for each damage state under anterior shear loading. (**A**) no damage (**B**) 2000 N Damage (**C**) ACL Removed.

**Figure 13:**
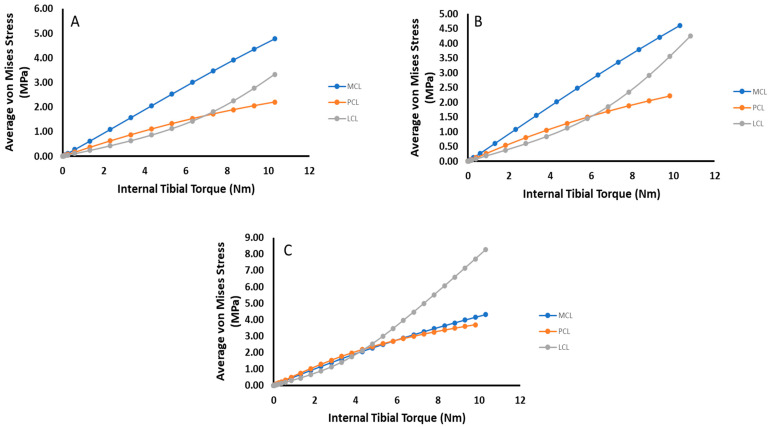
Comparative plots of the relative stress amounts for each ligament for each damage state under tibial torque loading. (**A**) no damage (**B**) 2000 N Damage (**C**) ACL Removed.

To further summarize, Figure 12 and Figure 13 compare the change in average von Mises stress between each ligament for each damage state and loading case. In both cases of anterior shear, the dominant loaded ligament changes over time, with the LCL rising in stress carried significantly as ACL damage occurs.

## 4. Discussion

In this work, the effects of localized ACL damage on its neighboring ligaments (MCL, PCL, and LCL) was investigated under anterior shear and internal tibial torque loading. This was performed by examining the changes in von Mises stress contour plots in the mid-substance of each ligament and the average mid-substance von Mises stress for different damage states. From this, it was found that the stress contours shifted in a non-homogenous manner, and that, in general, the average stresses increased with respect to ACL damage, though the MCL exhibited a flection point between increasing and decreasing in anterior shear loading and decreased under torque loading as damage increased. The results obtained in the present study give insights into how ACL damage may affect other ligaments. However, due to the variation of mechanical properties of the ACL, as well as constraints used in this model, it should be noted that this work speaks more to rends as opposed to exact real-world phenomena.

Investigations into the effects of ACL damage primarily focus on kinematics for fully damaged ACLs or by assuming the damage is uniform [11,12]. Works such as Lujan et al., Ellis et al., and Ochi et al. have demonstrated that deficiencies in the ACL can affect the neighboring ligaments, and vice versa [14,16,32]. The work of Ochi et al. is especially of note, as the deficiencies in the PCL lead to a significant change in tissue fiber diameter and density. As indicated prior, these works all assumed the deficiencies were caused by fully torn tissue, as opposed to mechanically compromised tissue via localized damage. As such, minimal work investigating the effects of localized ACL damage has been performed. From the results summarized in Figure 12, it is evident that the PCL carries the highest loading under anterior shear loading for no to moderate levels of damage (Figure 12A,B), though eventually the LCL overtakes the PCL, while the MCL does not drastically change (Figure 12C). In this manner, it is evident that in an average sense, the MCL’s risk to further damage under anterior shear loading has not changed by any significant measure, while the PCL and LCL have both increased. At 100 N, the average increase in von Mises stress within the MCL is about 5% between the undamaged and fully damaged cases. Comparatively, the results of the increase in strain within the MCL observed by the experimental work of Lujan et al. was at maximum around 4% [16]. For relatively small loadings, a one-to-one correlation between stress and strain should be expected due to the toe region of the stress-strain curve. In addition, similar jumps in magnitude and location of increase were observed by Ellis et al. using finite element modeling [32]. Therefore, while specific data increase in stress within the other ligaments is sparse, our results are still in line with what is available in literature.

The internal tibial torque data summarized in Figure 13 tell a similar story as their anterior shear counterpart. In this case, except for when complete tearing has occurred, the MCL carries the most loading and stays relatively constant, especially for torques below 5 Nm (Figure 13A,B). For complete ACL damage, the LCL takes the most loading for torques beyond 5 Nm but carries the lowest at torques below this threshold (Figure 13C). This shows that in the presence of moderate localized ACL deficiencies, the risk of damage to the MCL in an average sense has remained the same, while both the PCL and LCL have increased their risk.

Viewing these results, it may be apparent that for moderate localized ACL damage, the increased risk to the neighboring ligaments is relatively low. While likely true in an average sense, the changing stress contours may imply otherwise. For each ligament, the non-homogenous changes in stress contour carry interesting implications as to potential morphological effects in each ligament. It has been established that changes in the loads carried by the ligaments can lead to profound changes in the underlying microstructure of the ligament, as in the experimental work of Ochi et al. [14]. However, that study only looked at one location. The results of the present study indicate that over time, the ligaments will likely reorganize their microstructure non-homogenously. This will, in time, lead to instabilities in the joint overall, and, through the formation of new stress risers due to the mechanical integrity mismatch within the tissue, put the other ligaments at further risk of injury. Therefore, moderate localized damage to the ACL may spurn injuries to other ligaments. When the ACL is completely torn, the possibility for these changes becomes even more pronounced, particularly within the LCL for high anterior shear and tibial torque loadings. For this reason, it is evident that early intervention when ACL injury occurs will reduce the probability of additional complications in the other ligaments of the knee. Moreover, as the potential benefits and risks of ACL repair are still largely not well known, it is thereby recommended that regular assessment of the knee joint, especially if injury is suspected, should be performed to allow for early intervention [33,34,35,36,37].

## 5. Conclusions

A finite element model exploring the effects of localized damage within the ACL on stresses within the other neighboring ligaments was developed. Through this, it was observed that stresses generally increased within the neighboring ligaments as localized ACL damage increased, except for the MCL, which had a notable inflection point. Changing stress contour plots indicated the possibility of non-homogenous morphological changes within the tissue as ACL damage increases. At the same time, the increased loading on the PCL and LCL indicated higher risk of injury in those tissues post ACL damage, which further shows why early detection of ACL injury is important to begin treatment before additional complications can arise.

There are limitations in the present study that should be considered. This model did not include the meniscus, which may serve to impede some of the stress increases observed by taking on more of the loading within the meniscus. This model also used the same mechanical model for each tissue, and damage within the other ligaments aside from the ACL was not considered. This leaves out the potential for chain reactions of damage occurring once ACL damage occurs. In addition, data from one source from one strain rate were used to develop the model. Future models should endeavor to expand this, so that even more generalized cases can be examined.

The present work establishes that for localized damage to the ACL without complete tearing, the loadings on the neighboring ligaments can change significantly. It is thus important to establish methods for predicting ACL injury before complete tearing to avoid complications caused by reduction in mechanical integrity within the ACL, as localized damage spreads and eventually completely tears.

## Figures and Tables

**Table 1:** Summary of ligament’s material parameters.

*C*_1_ (Pa)	*D*	K_1_ (Pa)	K_2_	*k*	*λ*_0_	*λ_c_*	*β*
5,000,000	1 × 10^–9^	20,600,000	0.201	0	1.183	1.35	0.00019

## Data Availability

The data presented in this study are available on request from the corresponding author.
